# The Utility of Next-Generation Sequencing for Primary Immunodeficiency Disorders: Experience from a Clinical Diagnostic Laboratory

**DOI:** 10.1155/2018/9647253

**Published:** 2018-05-16

**Authors:** Atil Bisgin, Ibrahim Boga, Mustafa Yilmaz, Gulbin Bingol, Derya Altintas

**Affiliations:** ^1^Medical Genetics Department, Balcali Clinics and Hospital, Faculty of Medicine, Cukurova University, Adana, Turkey; ^2^Cukurova University Adana Genetic Diseases Diagnosis and Treatment Center (AGENTEM), Adana, Turkey; ^3^Division of Pediatric Allergy & Immunology, Faculty of Medicine, Cukurova University, Adana, Turkey

## Abstract

**Introduction:**

Primary immune deficiency disorders (PIDs) are a group of diseases with profound defects in immune cells. The traditional diagnostics have evolved from clinical evaluation, flow cytometry, western blotting, and Sanger sequencing to focusing on small groups of genes. However, this is not sufficient to confirm the suspicion of certain PIDs. Our innovative approach to diagnostics outlines the algorithm for PIDs and the clinical utility of immunophenotyping with a custom-designed multigene panel.

**Materials and Methods:**

We have designed a diagnostic algorithm based on flow cytometry studies to classify the patients; then the selected multigene panel was sequenced. In silico analysis for mutations was carried out using SIFT, Polyphen-2, and MutationTaster.

**Results and Discussion:**

The causative mutation was identified in 46% of PIDs. Based on these results, this new algorithm including immune phenotyping and NGS for PIDs was suggested for the clinical use.

**Conclusions:**

This study provides a thorough validation of diagnostic algorithm and indicates that still the traditional methods can be used to collect significant information related to design of most current diagnostics. The benefits of such testing are for diagnosis and prevention including the prenatal and preimplantation diagnosis, prognosis, treatment, and research.

## 1. Introduction

Primary immune deficiency disorders (PIDs) are a group of diseases where genetic basis of many has been identified in the last few years. The traditional diagnostics for PIDs have evolved from clinical evaluation, flow cytometric studies, or western blotting [1]. Immunodeficiency patients are classically evaluated due to the clinical presentation by initial screening tests including complete blood count (CBC), immunoglobulin levels, complement levels, specific antibody titers, and enumeration of lymphocyte subsets [2, 3]. Even though the traditional diagnostics are sufficient enough to confirm the suspicion of certain PIDs, molecular tests are still required to permit a conclusive diagnosis [4, 5].

Next-generation sequencing (NGS) has a great implementation on clinical diagnostics and research applications in recent years for Mendelian disorders such as PIDs. Those findings have increased our knowledge through novel gene discovery related to PIDs and refined phenotype-genotype correlations [6]. NGS has created its own potential to improve the diagnosis rate of PIDs by its reduced cost and time of testing as compared to standard clinical genetic testing [7, 8]. Thus, the clinics more focused on PIDs get the chance to accelerate the implementation of treatment and also to identify the causes of both typical and atypical cases, particularly for critical children who benefit from a significant reduction in morbidity and mortality [9, 10].

Genetic counselling together with clinical evaluation plays a critical role in the appropriate use of these tests, which have great potential to improve treatment outcomes [11, 12]. Our molecular diagnostic laboratory designed NGS multigene panels in association with specific PIDs that were classified by immunophenotyping. We describe our experiences within this manuscript, and the implementation of diagnostic testing including the immune phenotyping by flow cytometry followed by the next-generation sequencing of multigene panels in clinical use.

## 2. Materials and Methods

### 2.1. Demographic Features

All the patients referred to this study were selected from the south and south east part of Turkey. Consanguinity exists as first-degree cousins in the pedigree analysis of patients except the patients 8, 10, and 11. The mean age at the diagnosis is 9.6 (±2.4) months. The diagnostic algorithm for immune deficiency patients in our hospital includes immunophenotyping and molecular testing. Thus, all the newborns go for further laboratory investigations only if PID suspicion exists due to the family history, pedigree analysis, and clinical findings.

### 2.2. Immunophenotyping

Flow cytometry (Becton Dickenson FACSCalibur) was used to evaluate specific cell (sub)populations, cell surface, and intracellular proteins. Major forms of PIDS including severe combined immunodeficiency (SCID), agammaglobulinemia, hyper-IgM syndromes (HIGM), hyper-IgE syndromes (HIES), Wiskott-Aldrich syndrome, Mendelian susceptibility to mycobacterial disease (MSMD), and chronic mucocutaneous candidiasis (CMCD) can be effectively identified by immunophenotyping. Thus, flow cytometry is used in our diagnostic algorithm as a bridge for the choice of multigene NGS panels.

Flow cytometry application includes five steps: first the evaluation of specific cell (sub)populations for SCID (T, B, and NK cells), agammaglobulinemia (B cells), HIES, and CMCD (Th17 cells); second the evaluation of specific cell surface proteins for HIGM (CD40 and CD40L), MSMD (IFN-*γ*r1 and IL-12R*β*1), and X-SCID (CD132); third the evaluation of specific intracellular proteins for Wiskott-Aldrich syndrome (WASp), agammaglobulinemia (BTK), and DOCK8 deficiency (DOCK8); forth the evaluation of specific nuclear protein for IPEX (FOXP3); and lastly the evaluation of biologic effects as in HIGM (memory cells).

### 2.3. Collection of Blood Samples and DNA Isolation

The 37 blood samples from the patients who were referred to Departments of Medical Genetics and Pediatric Immunology, Faculty of Medicine, Cukurova University, in southern Turkey due to the immunodeficiency diagnosis were included in the study. Then, the genomic DNA was isolated by using the QIAamp DNA Blood Mini Kit (Qiagen, Hilden, Germany), according to the manufacturer's instructions. The quality of DNA samples was assessed by the Qubit™ Fluorometric Quantitation (Thermo Fisher Scientific, Waltham, MA, USA).

### 2.4. Next-Generation Sequencing (NGS)

The multigene panels were selected upon the flow cytometric results. As shown in Table 1, the multigene panels were also divided into groups according to the immunophenotype. All the genes in these multigene panels (listed in Table 1) were next-generation sequenced including all the coding exons, introns, and their flanking regions of at least 50 nucleotides upstream and downstream of each exon and 1 kb of both the 5′ promoter regions and the 3′ UTRs, using the QiaSeq targeted DNA panels (Qiagen, Hilden, Germany) that were customized. The 40 ng of genomic DNA was used to perform PCR reactions able to amplify the entire target region. These primers contained a universal adaptor sequence that is required for downstream sequencing reactions. All the libraries obtained were pooled and sequenced using the Next-Generation System (Illumina MiSeq, California, United States).

### 2.5. NGS Data Analysis

NGS sequence data were processed using the Mutation Taster Statistics software programme (http://www.mutationtaster.org/info/statistics) and CLC Genomics Workbench (Qiagen Bioinformatics, Germany) for analyzing, comparing, and visualizing the sequencing data. To evaluate the performance of all sequencing process, we have analyzed all the data by two different bioinformatics tools. More than that, quality control assessments were also performed by our bioinformatics specialists. Thus, a report/patient containing the sequence-coverage/exon and the list of high confidence variants could have been obtained. A minimum absolute coverage/exon of 200x is allowed and only variants present in both directions and with a minimum coverage of 25% default were contemplated in further analysis. The identified alterations were cross-referenced to the other samples within the same run as well as to the cumulative database. After filtering the known SNPs, we determine which alterations are most likely novel and warrant further consideration.

The novel mutations underwent the in silico analyses by using SIFT, Polyphen-2, and MutationTaster. All the parental testing processes were planned to identify the carrier status of the mother and father of the patients [13–15]. While SIFT and MutationTaster had only a single algorithm option for analysis, Polyphen-2 includes HumDiv and HumVar algorithms [14]. Thus, by using these three-program systems, we improved the accuracy of pathogenicity prediction over the use of any single program only when variants with relatively consistent predictions were analyzed.

All changes deemed of potential clinical relevance were confirmed by Sanger sequencing.

### 2.6. Ethical Statement

All procedures performed in this study were in accordance with the ethical standards of the institutional ethical and national research committee and with the Helsinki declaration.

## 3. Results and Discussion

For each of the 37 patients tested thus far, an average of 2 to 4 variants of unknown clinical significance were detected. Family studies were performed to define the pathogenicity of these changes together with the in silico analyses. After the exclusion of clinically irrelevant and benign/likely benign variants, the causative mutations were identified in 17 out of 37 patients with suspected PIDs (Table 2). Most interestingly the identified mutations in 5 out of 17 were novel mutations (patient numbers 1, 10, 14, 16, and 17). All these novel variants were confirmed by Sanger sequencing and the parental carrier testing.

There is significant cellular heterogeneity in the hematopoietic system, especially in the subsets circulating in blood. Immunophenotyping via flow cytometry and quantization of peripheral blood lymphocyte subsets have been shown to be either diagnostically and/or prognostically useful in several PIDs but not all. As in our study in cases, where the diagnosis based on immunological phenotype is not indicative, larger targeted gene panels could be assessed. However, relevant laboratory data always have to be correlated with the clinical phenotype.

Another possibility is that many of undiagnosed suspected patients (*n* = 20), who cannot be classified by immunophenotyping and immunoglobulin profiles, may have had as yet uncharacterized mutations in other genes. Thus, whole exome sequencing should be performed for further genetic etiologies.

Our results highlight the clinical potential of our algorithm via immune phenotyping and molecular testing. However, there is still a challenge in the field of genomics in that when potential novel genes/mutations or phenotypes are detected, functional studies and model systems are needed. Still, NGS systems via multigene panels in the diagnosis of clinically heterogeneous diseases became most successful diagnostic tool only in hands of experienced centers for detection of clinically relevant variants.

## 4. Conclusion

In summary, the availability of molecular genetic testing has profound implications for immunologists, patients, and their families. The benefits of genetic testing are for diagnosis including the presymptomatic and screening, prevention including the prenatal and preimplantation genetic diagnosis, treatment even including the gene therapy modalities, prognosis, and research.

Moreover, the application of molecular diagnosis by multigene NGS panels has broadened the understanding of PIDs. Molecular analysis together with conventional immunophenotyping thus is critical in modern disease classification, patient care, and management.

## Figures and Tables

**Table 1:** *PID Immunophenotype and multigene panels used*. Associated genes included in the multigene panels listed. Other well-known immune deficiencies such as ataxia telangiectasia, Nijmegen syndrome, Bloom syndrome, ICF syndrome, Wiskott-Aldrich syndrome, immunoosseous dysplasia, and Hoyeraal-Hreidarsson syndrome are not listed.

PID phenotype	Associated genes and multigene panels
T^−^B^+^NK^−^ SCID	*IL2RG, JAK3 *
T^−^B^−^NK^+^ SCID	*RAG1, RAG2, DCLRE1C, PRKDC, LIG4, NHEJ1 *
T^−^B^+^NK^+^ SCID	*IL7R, CD3D, CD3E, CD247, PTPRC, CORO1A, FOXN1, PNP *
T^−^B^−^NK^−^ SCID	*ADA, AK2 *
Agammaglobulinemia	*BTK, IGHM, IGLL1, CD79A, CD79B, BLNK *
Hyper-IgM syndrome	*CD40L, CD40, AICDA, UNG *
Hyper-IgE syndrome	*DOCK8, TYK2, STAT3 *
CVID	*TNFRSF13B (TACI), ICOS, TNFRSF13C (BAFF-R), CD40L, CD19, SH2D1A *
HPVsIDs	*TMC6, TMC8, RHOH, STK4 *
MSMD	*IL12B, IL12RB1, IFNGR1, IFNGR2, STAT1 *
SCN	*ELA2, HAX1, GFI1, MAPBP, WAS*
CD4+ T cell deficiency	*CIITA, RFX5, FRXAP, RFXANK, MAGT1, LCK, UNC119 *
CD8+ T cell deficiency	*TAP1, TAP2, TAPBP, ZAP70*

SCID, severe combined immunodeficiency; CVID, common variable immunodeficiency; CGD, chronic granulomatous disease; HPVsIDs, human papilloma viruses susceptibility immune deficiencies; MSMD, Mendelian susceptibility to mycobacterial disease; SCN, severe congenital neutropenia.

**Table 2:** *Patients', mutations list, and clinical indications*. 17 out of 37 patients were listed. The identified causative novel mutations (*n* = 6) marked in bold in patients 1, 10, 14, 16, and 17. All the other mutations were confirmed with the clinical data published by the references given. Clinical indications for molecular testing according to the family history, consanguinity status, and clinical presentation were given.

Patient #	Gene	Mutation	RS ID assigned by the dbSNP database/ClinVar accession #/somatic mutation database #	Clinical indication for molecular testing
1	*IL12RB1*	**p.C87 (c.261C>A) ** **homozygote**	**Novel**	(+) family history Consanguinity (+) BCG disease

2	*IL12RB1*	p.R175W (c.523C>T) homozygote	rs750667928	(+) family history Consanguinity (+) Mucocutaneous *Candida* infections

3	*IL12RB1*	p.R213W (c.637C>T) homozygote	rs121434494	(+) family history Consanguinity (+) Mycobacterial disease

4	*IL12RB1*	p.R175W (c.523C>T) homozygote	rs750667928	(+) family history Consanguinity (+) *Klebsiella pneumoniae*

5	*RMRP*	TIS+147G>T homozygote	rs753874439	(+) family history Consanguinity (+) Light-colored hair and malformed nails

6	*DOCK8*	p.E237K (c.709G>A) homozygote	rs11789099	(+) family history Consanguinity (+) Frequent pneumonia and hypereosinophilia

7	*DOCK8*	p.L284V (c.850C>G) homozygote	rs762990689	(−) family history Consanguinity (+) Hypereosinophilia

8	*STAT1*	p.E638Q (c.1912G>C) homozygote	COSM1014147 (MU1911384)	(−) family history Consanguinity (−)Candidiasis

9	*PNP*	p.Glu89Lys (c.265G>A) homozygote	rs104894453	(+) family history Consanguinity (+) Developmental delay and chronic diarrhea

10	*STAT3*	**p.R382Q (c.1145G>A) ** **heterozygote**	**Novel**	(−) family history Consanguinity (−)Dental abnormalities and hyper-IgE

11	*STAT3*	p.F621L (c.1863C>G) heterozygote	SCV000590715	(−) family history Consanguinity (−)Recurrent skin infections and hyper-IgE

12	*ATM*	p.V835S (c.2502_2503insA) homozygote	rs587779822	(+) family history Consanguinity (+) Ataxia

13	*ATM*	p.R35 (c.103C>T) homozygote	rs55861249	(−) family history Consanguinity (+) Ataxia

14	*ATM*	**p.D2708E (c.8124T>A) ** **homozygote**	**Novel**	(+) family history Consanguinity (+) Ataxia and elevated alpha-fetoprotein

15	*HAX1*	p.W44X (c.130_131 insA) homozygote	SCV000025090	(+) family history Consanguinity (+) Neutropenia

16	*DCLRE1C*	**p.T52M ** **(c.155C>T) ** **homozygote**	**Novel**	(+) family history Consanguinity (+) Leukopenia and low antibody levels

17	*DCLRE1C*	**IVS5-1G>A ** **homozygote**	**Novel**	(+) family history Consanguinity (+) Leukopenia and low antibody levels

RMRP, RNA component of mitochondrial RNA processing endoribonuclease; *PNP, *purine nucleoside phosphorylase.
